# The internal mammary artery – use as a free graft in coronary artery bypass grafting – evidence, technical considerations and controversies

**DOI:** 10.1177/02676591251393446

**Published:** 2025-11-06

**Authors:** Manoraj Navaratnarajah, Fadi Ibrahim Al-Zubaidi, Shahzad G. Raja

**Affiliations:** 1 Department of Cardiac Surgery, 111988 St Thomas Hospital, London, UK; 2 Department of Cardiac Surgery, John Radcliffe Hospital, Oxford, UK; 3 Department of Cardiac Surgery, 156725 Harefield Hospital, Middlesex, UK

**Keywords:** coronary artery bypass grafting, mammary arteries, arterial grafting, treatment outcome, cardiac surgical procedures

## Abstract

**Background:**

In-situ internal mammary artery (IMA) grafting remains the gold standard in coronary artery bypass grafting (CABG), particularly for left anterior descending artery revascularisation. However, the role of free-IMA grafts—especially free right IMA (RIMA) and select cases of free left IMA (LIMA)—has expanded in response to anatomical and technical constraints. This narrative review synthesises current evidence on free-IMA use during CABG.

**Methods:**

A structured literature search was conducted using PubMed (1946–2025) and Embase (1974–2025), supplemented by Web of Science, Google Scholar, and thesis repositories. Studies were included if they reported outcomes related to free-IMA grafting, regardless of pump status or harvesting technique. Of 74 eligible studies, 9 chosen studies specifically reported free-RIMA outcomes and were analysed in detail.

**Results:**

Free-RIMA grafting demonstrated excellent long-term patency (up to 96%) and favourable survival outcomes when used as composite or direct aorto-coronary grafts. Multi-arterial grafting (MAG) and total arterial grafting (TAG) strategies incorporating free-IMA conduits were associated with reduced major adverse cardiac events (MACE) and improved freedom from repeat revascularisation. Despite these benefits, uptake of free-IMA techniques remains low in Europe and North America, often limited by institutional preferences and operator experience.

**Conclusion:**

Current evidence supports the selective use of free-IMA grafts in CABG, particularly when in-situ deployment is not feasible. Prospective studies are needed to validate long-term outcomes beyond 10 years, compare free-IMA with radial artery grafts, and define optimal arterial configurations for durable revascularisation.

## Introduction

Survival and long-term major adverse cardiac events (MACE) are influenced by conduit choice during coronary artery bypass grafting (CABG).^1,2^ Complete revascularisation and arterial grafting improve long-term CABG outcomes, and supremacy of the internal mammary artery (IMA) is established.^3^ The landmark study by Loop in 1986 established the use of in-situ left internal mammary artery (LIMA) graft to left anterior descending (LAD) artery as the CABG gold standard.^4^ To augment benefits of LIMA use, growing focus upon increasing arterial conduit use during CABG, including the right internal mammary artery (RIMA) and radial artery (RA) has occurred. The RIMA displays similar structural and biological properties to the LIMA,^5,6^ showing added clinical benefit when used as free graft, or in-situ alongside the LIMA.^7–9^ Various arterial configurations are employed during multi-arterial grafting (MAG) and total arterial grafting (TAG),^10,11^ and their influence on graft patency and clinical outcomes is well studied.^6,10,11^ This knowledge encompasses the effects of free-RIMA compared to in-situ RIMA graft use, the former demonstrating excellent long-term patency in various anatomical contexts.^12–14^ In contrast, long-term outcomes and patency of free-LIMA graft are not well documented. Occasionally the LIMA pedicle is too short for use, is damaged whilst harvesting, or displays inadequate flow secondary to subclavian artery (SCA) stenosis. If technical problems are limited to the proximal LIMA, it remains a good conduit as a free graft.

While the majority of free-IMA grafts discussed refer to the RIMA conduit, free-LIMA grafting is also addressed in this review. Although in-situ LIMA remains the preferred conduit for LAD revascularisation due to its excellent long-term patency, free-LIMA use is occasionally required when in-situ deployment is precluded by the aforementioned situations. These scenarios necessitate alternative strategies to preserve arterial grafting integrity. This narrative review analyses the evidence relating to free-IMA use during CABG, particularly in scenarios where in-situ IMA grafting is not feasible due to pedicle injury, inadequate length, or SCA stenosis. Understanding the outcomes and configurations of free-IMA grafting is essential to guide intra-operative decision-making and optimise arterial revascularisation strategies. The review also addresses associated technical considerations, concerns, and controversies.

## Methods

Articles were identified through a literature search of English language articles in PubMed (1946 to May 25, 2025) and Embase (1974 to May 2025), last updated May 25, 2025. The recruitment period for included studies ranged from 1970 to 2022, based on availability of reported data. The search strategy focused on identifying articles studying use of internal mammary artery during CABG. Search terms included these key terms and their combinations: coronary artery bypass grafting, internal mammary artery or internal thoracic artery (left, right, bilateral, free or in-situ), graft patency, and arterial revascularisation. Although the search spanned 1946 to May 2025 (PubMed) and 1974 to May 2025 (Embase), relevant studies meeting inclusion criteria were published between 1985 and 2025. This yielded 427 publications from 1986 to 2025, of which 74 English language articles were included. Non-English studies were excluded to ensure consistency in interpretation and critical appraisal across the review team, and to minimise potential translation bias given the technical nature of surgical terminology and outcome reporting.

**Figure 1.**
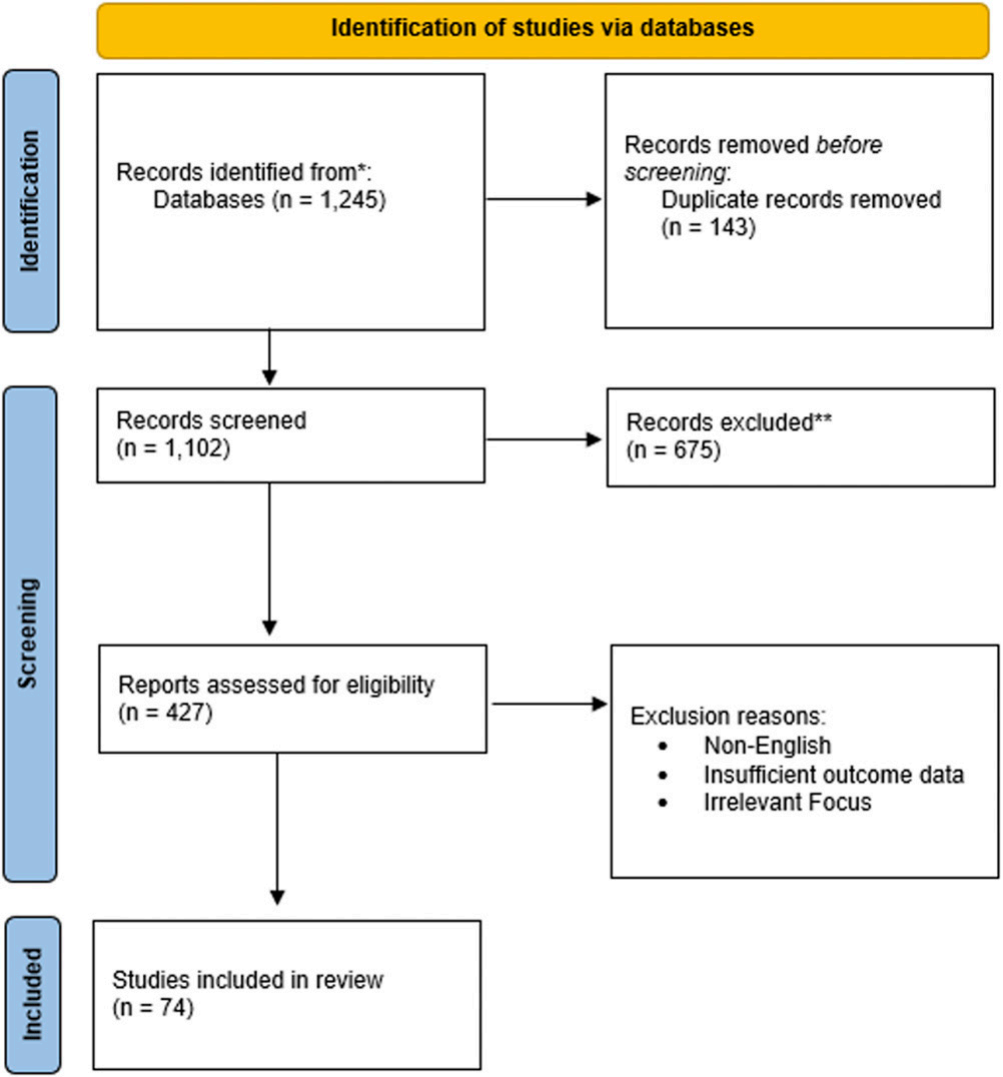
PRISMA flow diagram illustrating the literature search and study selection process.

In addition to PubMed and Embase, supplementary searches were conducted using Web of Science, Google Scholar, and thesis repositories. No additional eligible studies were identified beyond those captured in the primary search. The following representative search terms and Boolean operators were used: (“internal mammary artery” or “IMA” OR “LIMA” or “RIMA”) and (“free graft” OR “aorto-coronary” OR “composite graft” OR “Y-graft”) and (“coronary artery bypass” or “CABG”). Filters were applied to include human studies, full-text articles, and English-language publications. Two reviewers (M.N. and S.G.R.) independently evaluated and selected studies based on quality and relevance. Conflicts in study selection were resolved by mutual agreement or discussion with the co-author. Of the 74 included studies, 9 chosen studies specifically reported outcomes related to free-RIMA grafting (Figure 1). The majority of remaining studies contributed contextual or comparative data relevant to arterial grafting strategies, often not clearly isolating free-RIMA outcomes. Studies were eligible regardless of whether CABG was performed on-pump or off-pump. This approach reflects the procedural diversity in clinical practice and acknowledges that free-IMA grafting is employed across both modalities. No restriction was applied based on IMA harvesting technique. Studies were eligible regardless of whether the conduit was harvested as pedicled, skeletonised, or semi-skeletonised, provided outcomes related to free-IMA grafting were reported. This reflects the variability in surgical technique across institutions and enhances the generalisability of findings.

A free-IMA graft refers to an IMA conduit that is harvested and detached proximally from its native origin, then anastomosed to the ascending aorta or another conduit to provide inflow for coronary revascularisation. This technique is employed either when in-situ use is not feasible due to pedicle injury, inadequate length, or SCA stenosis or as an adjunct to an in situ IMA conduit.

Outcome measures assessed across included studies comprised graft patency (early and late), long-term survival, freedom from major adverse cardiac events (MACE), and need for repeat revascularisation. Patency was typically evaluated via postoperative coronary angiography or computed tomography angiography, with survival and MACE tracked through institutional or registry follow-up. Surgeon experience was not used as an inclusion criterion, as most studies did not report operator seniority or minimum experience thresholds. This limitation is acknowledged, and future studies should consider reporting operator-level data to better contextualise outcomes, particularly for technically demanding grafting strategies.

As this is a narrative review, formal risk of bias or quality assessments were not conducted. The focus was on synthesising existing evidence qualitatively rather than quantitatively pooling data, which would require systematic review methodology and standardized appraisal tools.

## Results

### History of free internal mammary artery use for CABG

**Figure 2.**
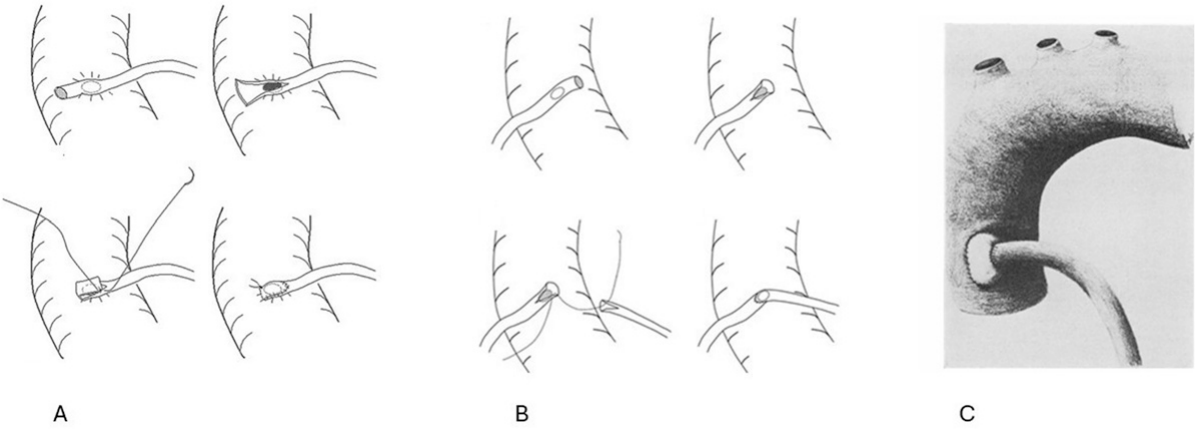
Proximal free-IMA to aorta anastomosis techniques. (A) Foldback technique depicted by Ito^19^, (B) Piggyback technique depicted by Hayashi^20^ and (C) saphenous vein patch depicted by Vidne^15^ (C).

**Figure 3.**
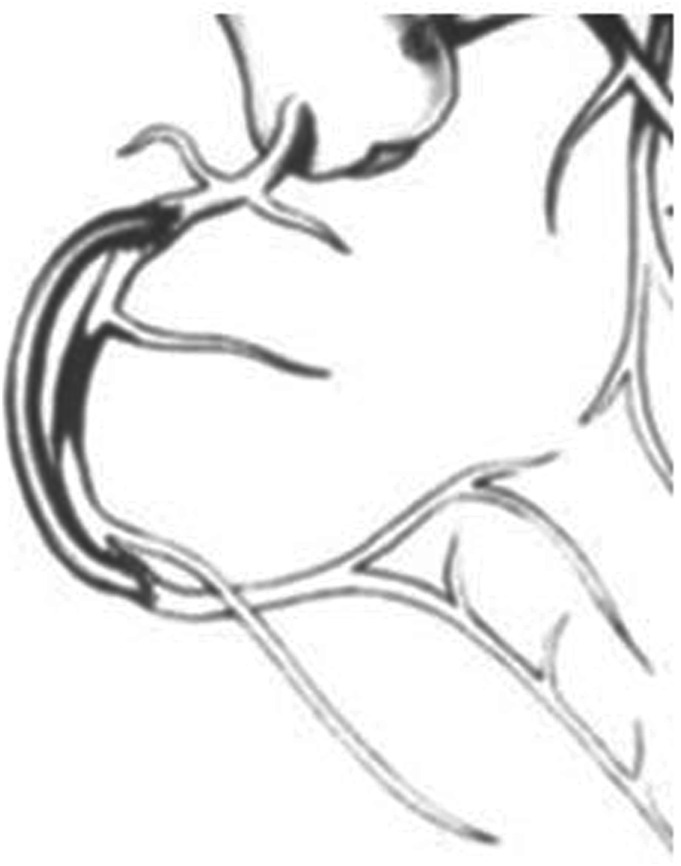
Coronary to coronary anastomosis depicted by Nottin^23^.

Use of free-IMA grafts for coronary revascularisation is not new. Reports from 1970s,^15–18^ show good clinical and angiographic results. Vidne described a 77-patient study in which 125 free-IMAs were grafted to left and right coronary targets using proximal vein-patch technique to ascending aorta^15^ (Figure 2). They reported excellent short-term results with resolution of symptoms. Schimert showed 100% angiographic free-IMA patency at 2 weeks-6 months in 25 patients using similar vein-patch technique to aorta.^16^ Loop in 1972 described a small patient series undergoing aorto-coronary bypass using free-IMA with a direct anastomosis,^17^ and Kanter described an elegant technique for aorto-coronary bypass with free-IMA using autologous pericardial patch.^21^ The use of free-IMA grafts then spread to coronary-coronary bypass grafting, with successful reports from the 1980s onwards.^22–27^ This technique involved bypassing a coronary vessel stenosis with a free-IMA or free-IMA segment, with proximal anastomosis originating from the same diseased vessel, or another coronary vessel (Figure 3). Nottin examined 143 patients undergoing CABG between May 1989 to December 1995.^23^ 138 patients received single, and 5 patients received double coronary-coronary bypass grafts in addition to other bypass grafts. 90.5% of coronary-coronary bypass grafts were performed on the RCA and like other series,^22–24^ proximal implantation site was predominantly the RCA origin (92.6%). A small number of coronary-coronary grafts were on LAD (2.7%), and between 2 different coronary arteries (3.3%). Early postoperative angiography showed 98.6% patency rate and exercise testing was normal at 3 years in 93%. Importantly, after 7-years, progression of coronary disease at proximal anastomosis site was never observed. A later 48-patient Texas Heart Institute study re-emphasised free-IMA coronary-coronary grafting safety, with 100% angiographic patency rate (n = 24) at mean follow-up of 16.5 months.^22^

### In-situ left internal mammary artery versus free left internal mammary artery

Limited data comparing free-LIMA versus in-situ-LIMA outcomes during CABG exists. Rates of free-LIMA use in published series are low at 2-3%.^28^ Long-term patency of in-situ LIMA graft is excellent reported at 93% at 10 years and 88% at 15 years in contemporary reports.^29,30^ Vistarini performed a retrospective propensity-score-matched analysis of 2 cohorts of 222 patients each, comparing free-IMA versus in-situ grafting to the LAD.^30^ The LAD was bypassed with a free-LIMA in 94.1%, and in-situ-LIMA in 99.2% respectively, the remainder receiving free or in-situ RIMA to LAD.

Reasons for free-LIMA use showed harvest injury in 50%, SCA stenosis in 43%, and inadequate in-situ LIMA length responsible in 13%. Cardiopulmonary bypass time was 14 min longer in free-IMA group. Post-operative adverse events were similar between cohorts; but higher rates of pulmonary infection, transfusion and renal failure occurred in free-IMA group. Free-IMA proximal anastomosis was either directly to ascending aorta (49%), or saphenous vein hood (51%).

Mean follow-up was 9.5 years and 5, 10- and 15-years survival were 86.7%, 71.2% and 53.7% in free-IMA group, and 91.4%,77.9% and 62.5% in in-situ IMA group. Risk of late death was similar, but absolute risk of hospital cardiovascular readmission was 7% higher in free-IMA group (54.5% vs 47.3%), although repeat revascularization rate was not different (18.4% vs 13.5%).

Authors concluded that CABG survival was not negatively influenced by using free-IMA to LAD, and survival benefit of in-situ LIMA to LAD grafting not compromised. However, they acknowledged the retrospective nature, use of 2 different free-IMA proximal anastomosis techniques, short median follow-up and lack of graft patency assessment as study limitations. They acknowledged at 10 years group survival curves diverged and due to patient follow-up attrition, their ability to characterise free-IMA to LAD effectiveness at late time points was limited, advocating longer follow up to confirm extended free-IMA durability.^30^

### In-situ right internal mammary artery versus free right internal mammary artery

RIMA has not received as widespread use as its left-sided opposite number during CABG, and use is restricted to the minority of surgeons routinely performing MAG or TAG. Patency rates are reported between 80 and 90% at 10 years.^31,32^ Compared to the free-LIMA, free-RIMA efficacy has been studied more, and is better understood. Several factors potentially influence RIMA patency: including grafted coronary location and disease severity, harvesting technique, free versus in-situ utilisation, and configuration. It is rational to expect a physiologically identical conduit to achieve similar excellent long-term patency as its colleague the LIMA if used in a similar fashion.

Excellent long-term angiographic study by Tatoulis demonstrated overall RIMA patency of 90% at 10 years, with patency to the LAD of 95% at 10 years, and 90% at 15 years.^32^ This group showed identical 10-years LIMA and RIMA patency to LAD (95% vs 96%). In-situ RIMA and free-RIMA demonstrated similar 10-years patency rates (89% vs 91%). In 1997 Tatoulis had laid the platform for the safety, versatility and expanded use of the RIMA as a free graft. 1454 patients received free-RIMA graft between 1986 and 1996 during CABG.^12^ In 99% free-RIMA was used alongside a LIMA graft with the proximal anastomosis placed directly on the aorta in the majority (n = 1441). Free RIMA was grafted to obtuse marginal arteries in 49.5%, posterior descending artery (PDA) in 19.7%, diagonal or intermediate in 11.8%, LAD in 8.1%, and right coronary artery (RCA) in 7.9% of patients. 5- and 7-years survival was excellent, 96% and 94% respectively. 71 patients with a free-RIMA underwent angiography at mean interval of 41.5 months for symptoms recurrence. In these patients 94.5 % of free-RIMAs were widely patent, 3 displayed a string sign, and 1 was occluded. Graft failures were all early (<1 year) and occurred where free-RIMA was grafted to a moderately stenotic RCA. Free-RIMA patency was similar to in-situ LIMA patency studied at a similar time interval.

The same group retrospectively studied symptom-directed angiograms in 1434 patients undergoing CABG between 1982 and 2002. 1482 LIMA, 307 free-RIMA and 317 in-situ RIMA grafts were studied with mean re-angiogram interval of 80 months.^33^ 82% of patients received LIMA to LAD and RIMA was placed to RCA in 40% and circumflex artery in 35% respectively. 96% of LIMA and 88% of RIMA grafts were patent with graft failure defined as ≥80% stenosis. RIMA patency to LAD or left-sided coronary targets was identical to that of LIMA. Worst RIMA patency occurred when grafted upon the RCA, and superior patency was achieved with free-RIMA proximal anastomosis to aorta, compared to in-situ RIMA to the RCA. Authors concluded that even in patients with adverse symptoms, excellent LIMA and RIMA long-term patency is achievable, remaining stable through all time intervals. (Table 1)

**Table 1.** Summary of chosen studies reporting outcomes specific to free-RIMA grafting during CABG.

RIMA study outcomes
Overall 10-years RIMA patency (80–90%)^28,30,32,34^
Overall long-term RIMA patency is equivalent to LIMA patency when grafted to LAD^12,30,33^
Free-RIMA patency is equivalent to in-situ RIMA patency at 10 years^30,32^
Free-RIMA patency is equivalent to in-situ RIMA patency when grafted to left coronary system^30,32^
RIMA patency to the RCA is lowest^33^
Free-RIMA patency is superior to in-situ RIMA when grafted to RCA^33^
Free-RIMA Y-graft patency and in-situ RIMA graft patency appear equivalent during BIMA grafting^35–37^
Severity of target vessel stenosis is the predominant factor influencing free-RIMA patency^30^

Of the 74 studies included in the review, 9 chosen studies provided isolated data on free-RIMA configurations and outcomes.

### Right internal mammary configurations during multi-arterial grafting and total arterial grafting

**Figure 4.**
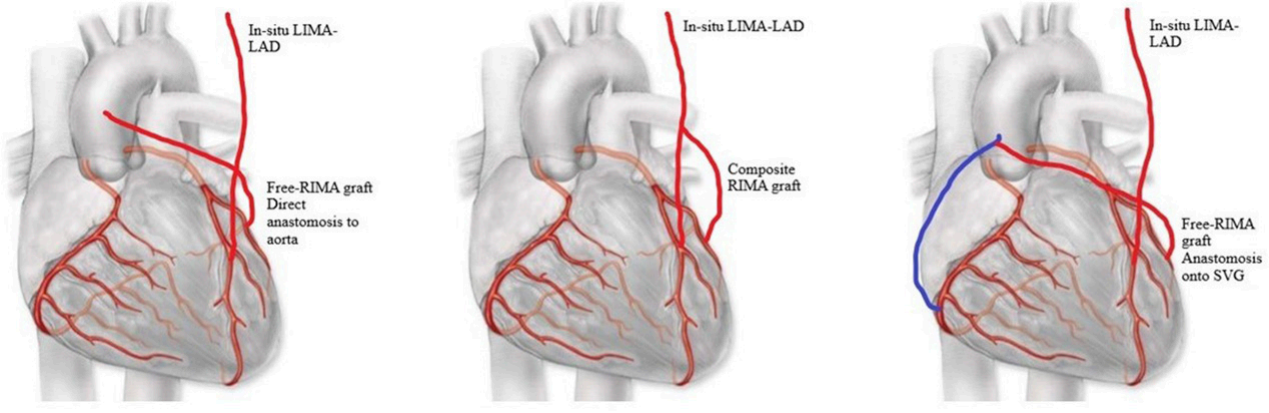
Different free-RIMA graft configurations.

The RIMA is utilised in various CABG configurations, including in-situ RIMA to the left or right coronary system, free-RIMA with direct proximal anastomosis to aorta, vein hood or radial artery hood, or free-RIMA to the LIMA as composite graft Y or T-graft (Figure 4).

Calafiore studied 1818 patients undergoing CABG using bilateral IMAs (BIMA). 1378 patients received in-situ IMAs and 440 received Y-grafts using free-RIMA.^34^ 30-days outcomes and 8-years survival (95.8% vs 94.8%) were equivalent between groups. 295 patients underwent early, and 88 patients late angiographic interrogation. Early and late patency rates were similar, being 100% and 99.2% respectively at the latter timeframe, with a mean follow-up of 17 months.

This finding that RIMA patency appears independent of in-flow configuration is supported by Cleveland clinic data.^28^ 7092 patients underwent BIMA grafting between 1972 and 2016. 1331 patients receiving BIMA grafts with post-op angiography were studied: including 835 (63%) in-situ RIMAs and 496 (37%) free-RIMAs and 1331 LIMAs. Patients underwent angiography for various reasons. Of the patients receiving free-RIMA grafts 63% were anastomosed directly to aorta, 20% to in-situ LIMA and 17% to saphenous vein graft (SVG), or RA hood. 98% of LIMAs were used in-situ, with 20 (2%) utilised as free-LIMA; 16 anastomosed to aorta, 4 to either the SVG or RA graft.

15-years overall RIMA patency was good with various inflow configurations; 91% in the in-situ configuration, 91% in free-graft configuration anastomosed to aorta, and 89% in free composite graft to LIMA. Un-adjusted analysis of free-RIMA patency off SVG hood had significantly lower patency (77%), but this difference disappeared after adjusting for coronary target location and stenosis severity. Like other studies no difference in patency was found between RIMA to LAD and LIMA to LAD, and RIMA to LAD had better patency than RIMA to non-LAD targets. After adjusting for target location, free-RIMA and in-situ RIMA patency were similar.

Prospective randomised control trial (RCT) by Glineur compared in-situ RIMA with free-RIMA Y-graft angiographic patency in 304 patients during BIMA grafting, finding no difference at 6-months with 97% patency.^35^ At 3-years angiographic follow-up including 75% of the original population, again, no difference in patency of in-situ RIMA (93%) and free-RIMA Y-graft configuration (94.5%) was seen.^36^ However, compared with in-situ configuration, free-RIMA Y-grafting configuration displayed lower 7-years MACE rate. Similar patency findings were demonstrated by Hwang during off-pump CABG using BIMAs; showing no difference between in-situ RIMA and free-RIMA Y-graft angiographic patency at 5-years, or clinical outcomes/survival at 10 years.^37^ Yanagawa performed a meta-analysis of 8 studies concluding that both grafting strategies broadly offered similar clinical benefits, but that more long-term prospective trials were needed.^38^

However, certain studies show differing differences between configurations. Mazrouk showed that in-situ RIMA group displayed better late survival than free-RIMA group during MAG, with similar hospital readmission and repeat revascularization rates.^39^ Hayashi in another small retrospective study showed free-RIMA to have better 5-years angiographic patency than in-situ RIMA, with no survival difference.^20^ More recent retrospective 4-centre, 1171-patient study by Hayashi showed free-RIMA to circumflex to have superior 4-years angiographic patency, and 10-years survival and MACE rate, compared to in-situ IMA (R/L) to circumflex configuration.^40^

### Biological properties and functional characteristics of the internal mammary artery

#### Histological structure

The IMA is the only predominantly elastic human artery, composed of an intima with a well-formed internal elastic lamina and a media formed by interconnected elastic lamellae.^41^ The elastic layer within the media protects against atherosclerosis by prevention of smooth muscle infiltration towards the intima. Paucity of media smooth muscle limits the proliferative response to mechanical stretch and growth factors, thus also ameliorating vasospasm.^42,43^ The IMA was initially classified as elastic, but various histological patterns have now emerged. Most patients possess an elasto-muscular media in proximal and distal IMA segments, with the mid-segment remaining mainly elastic.^41^

#### Biological properties

The IMA has a unique conduit feature of an endothelium producing significant quantities of vasodilators.^44^ This enhances endothelium-dependent relaxation and protects against vasospasm and promotes positive long-term remodelling.^45,46^ IMA-endothelium-produced vasodilators underlie protective vessel effects; retarding coronary artery disease progression via mainly anti-atherosclerotic mechanisms.^47,48^ Another property driving excellent patency is the IMA’s remodelling capability, with increased diameter and flows occurring over time.^49–51^ These endothelial-dependent and structural properties are believed drivers of superior IMA graft function over venous conduits; although the difference in endothelial-derived relaxation between other arterial conduits is less marked.^45^

#### Functional studies - Free-IMA versus in-situ IMA

Concerns existed regarding whether IMA transection and resulting denervation, loss of vasa vasorum and lymphatics, affected vasodilator reserve and endothelial function.^5,52^ Professor Sir Magdi Yacoub’s team compared endothelial-dependent/-independent vasodilatation in free and in-situ IMA grafts. No significant difference was seen at mean 2-years angiographic follow-up with all grafts patent.^5^ They concluded that free-RIMA dilatory capability was unaffected by transection, functioning almost identically to its pedicled counterpart. Similarly, Glineur demonstrated endothelium-dependent and endothelium-independent vasodilator capacity of the two branches of a Y-graft IMA configuration appeared similar at 3 years; reenforcing the idea that preserving an in-situ IMA was not essential to preserve endothelial function or vasodilator reserve.^52^

Functional studies comparing free-RIMA with in-situ RIMA during BIMA grafting are not plentiful. When BIMA is performed to left coronary system, no difference in intraoperative graft flow is demonstrated between LIMA-LAD and RIMA-LAD grafts. An identical result was seen when free-RIMA Y-graft configuration flow was compared to direct free-RIMA flow to ascending aorta, when grafting diagonal or circumflex vessels.^53^ Neragi-Miandoab demonstrated in the acute peri-operative phase, aorta-free-RIMA anastomosis displayed higher mean conductance and fractional flow than composite T-graft, but no clinical differences were seen.^54^ Sakaguchi showed superior flow reserve in favour of dual inflow free-graft to aorta configuration over composite Y-graft, where RA and RIMA were used as free grafts.^55^ Hayashi have shown free-RIMA displaying superior 5-years patency than in-situ despite similar intra-operative flow parameters.^20^ Therefore, the exact long-term clinical relevance of intra-operative and short-term functional flow assessments requires definition.

### Free right internal mammary artery versus radial artery

Mean luminal diameter is smaller in IMA than RA. IMA media is thinner, has fewer muscular fibres and internal elastic lamina discontinuities than RA.^56^ The RA is considered more muscular and less elastic than IMA, less resistant to reactive oxidative species, to produce less nitric oxide and more susceptible to intimal atherosclerosis.^57^ Therefore, it is reasonable to expect free-RIMA to display superior long-term results than a free-RA when grafted within a similar context.

Whether free-RIMA is superior to free-RA is still debated. Few RCTs directly compare angiographic RA versus RIMA outcomes. Radial Artery Patency and Clinical Outcomes (RAPCO) trial compared clinical outcomes and patency of RA versus free-RIMA and RA versus SVG. 10-years follow-up showed RA patency was significantly higher than free-RIMA (89% vs 80%), and patients receiving RA showed superior long-term survival (91% vs 84%). In this excellent single-centre study all patients received in-situ LIMA to LAD, and RA, RIMA or SVG to most important non-LAD target, with all second conduits used as aorto-coronary grafts. However, meta-analysis including 2780 patients, RIMA (*n* = 145), RA (*n* = 871) and SVG (*n* = 845) showed angiographic superiority of RIMA and RA over SVG, concluding that the RIMA is expected to achieve better patency than RA, but further studies were needed. Observational studies utilising various RIMA configurations show both RIMA superiority over RA^58^ and equipoise^59^ regarding survival and clinical outcomes. A meta-analysis of propensity scored matched patients showed RIMA use compared with RA was associated with superior long-term survival and freedom from repeat revascularization.^60^ Importantly, added clinical benefit when BIMA is combined with RA graft, over BIMA + SVG has been demonstrated.^61^

This suggests that RA and RIMA are both excellent conduits, both superior to SVGs and their roles should be “complementary not competitive”.^62^ The choice is not simple. The left RA is harvested simultaneously with LIMA and more time-efficient, and less delicate, less prone to injury compared to skeletonized RIMA. RA is long allowing reach to almost any target as aortic free-graft, and all targets as a LIMA Y- or extension graft. However, spasm prophylaxis, and avoidance of competitive flow are mandatory. The RA is preferred in patients with elevated risk of sternal wound complications, COPD, older age and where in-situ or free-RIMA length is inadequate. Like the IMA the RA shows continued excellent long-term patency once early patency is established, displaying a patency ladder; highest to LAD, then diagonal, then circumflex, and least to RCA/PDA, with patency unaffected by harvest method or inflow source.^32,62,63^ RA patency appears similar to RIMA and LIMA, when anastomosed to similar vessels.^32,62–64^

### Revascularisation guidelines and technical considerations

#### Guidelines

Current guidelines advocate that unless in rare circumstances, all CABG patients should receive at least one IMA, grafted preferentially to the LAD (Class I). An additional arterial graft should be considered depending on patient life expectancy, risk factors for sternal wound complications, coronary anatomy, target vessel stenosis, and surgeon expertise^65,66^ (Class II A). BIMA grafting should be considered in patients not at high-risk of sternal wound infection (Class II A). The various configurations for arterial grafting using BIMAs are not commented upon, and no recommendations given relating to conduit inflow patterns in the guidelines. This is due to lack of prospective randomised controlled long-term evidence examining the effects of inflow configurations on graft patency and outcomes. The crucial influence of target vessel stenosis severity on graft patency is established, and lesion sets are often imperfectly controlled for, thus limiting external comparison or generalisability of studies. Therefore, the ideal BIMA configuration remains debateable as is the best anastomosis site/technique for a free-IMA graft. There is limited evidence directly comparing free-IMA graft efficacy when anastomosed to aorta, vein hood or radial artery hood with T/Y--graft configuration where free-IMA joins in-situ LIMA. Overall, the studies discussed earlier show no major differences when comparing in-situ RIMA with free-RIMA T/Y-graft composite technique.^35–37^

#### Technical recommendations

When constructing a proximal aortic anastomosis for free-IMA the thin-walled IMA must not be flattened when suturing to the thick-walled aorta. The best anastomotic technique is not established and good results are obtained with either direct anastomosis, or via vein hood or RA hood onto aorta^11,15,16,30,32,62,67,68^ (Figures 2 and 4). Proximal anastomosis can also be onto another SVG or RA graft, but the grafted target outflows and likelihood of competitive flow between territories must be considered. In general, it is recommended to make a small aortic defect with a 3.0–3.5 punch and perform the anastomosis with aortic cross-clamp applied.^30,32^ To avoid anastomosis flattening during free-arterial grafting, making the conduit aperture 30% larger than aortic aperture is advised.^32,62^ Early graft failure is thought largely related to sub-optimal proximal anastomotic technique.^11^ Hayashi compared 2 proximal free-RIMA techniques finding similar 5-years patency (96% vs 100%), survival (93% vs (90%) and MACE rate between “piggyback” and “foldback” techniques^20^ (Figure 2). Free-RIMA intra-operative graft flow was similar between groups. Excellent short and medium-term patency rates are demonstrated using both the “piggyback”,^40,69^ and “foldback” techniques^19,40^ (Figure 2). In agreement with previous authors, to optimise free-IMA patency, we feel a key concept during arterial grafting is that the reach of an IMA should not decide its target, more so the intended target should dictate the IMA configuration, with strict attention paid to outflow configurations.^28^

### Free LIMA

Use of a free-LIMA is usually unplanned during CABG and rates of use are universally low. The technical considerations described for free-RIMA also apply to a free-LIMA graft. An often-overlooked principle is anticipated use of a free-LIMA. Routine screening for SCA stenosis is not performed and is advocated by some.^30^ Pre-operative measurement of bilateral arm non-invasive blood pressures, doppler ultrasound examination of supra-aortic vessels and invasive SCA angiography at catheterisation are all useful.^30^ Reluctance for screening is fuelled by low rate of SCA stenosis in CABG patients and may be better reserved for higher risk individuals, such as patients with peripheral vascular disease or previous radiotherapy.

If in-situ LIMA graft use is prevented by injury or inadequate length, consideration must be given to RIMA harvesting, and the BIMA configurations described earlier borne in mind, even if not originally planned. Maximal length/lengths of useable LIMA should be obtained even if thought inadequate for constructing a direct proximal free-LIMA-aortic anastomosis. This attitude will facilitate composite arterial grafting to the left system and is preferable to non-use of either IMA. Particularly in a young patient, in a patient not unsuitable for BIMA, efforts should be made to achieve maximal arterial grafting to the left coronary system, with an IMA-LAD graft.

### Sequential grafting

Most evidence relating to sequential IMA patency relates to in-situ IMA,^70^ and is mainly derived from observational studies, and suggests similar patency of sequential IMA grafting to that of individual IMA.^70^ Design of anastomosis (diamond vs parallel) and target vessel stenosis severity are critical determinants.^71^ Free-RIMA sequential grafting is performed less commonly and evidence is limited. Fukui in a retrospective study demonstrated that sequential free-RIMA grafting showed identical early and late angiographic patency and clinical results to free-RIMA single-target grafting. Free-RIMA in-flow was 27.4% from aorta and 72.6% from other artery in the individual group, and free-RIMA inflow was solely from other grafts in the sequential group.^72^ 1-year patency rates were similar at 93.0% and 91.2% respectively.^72^

## Discussion

The evolving role of free internal mammary artery (IMA) grafting in coronary artery bypass grafting (CABG) reflects a broader shift toward more anatomically tailored and technically versatile revascularisation strategies. While in-situ IMA grafting remains foundational, the selective use of free-IMA conduits—particularly free right IMA (RIMA)—has enabled surgeons to overcome anatomical constraints and expand arterial reach. This flexibility is especially valuable in patients with hostile mediastinal anatomy, prior sternotomy, or complex coronary targets. The evidence reviewed underscores that free-IMA grafts, when deployed judiciously, can preserve the long-term benefits traditionally associated with in-situ IMA use.

Despite these advantages, the uptake of free-IMA techniques remains limited in many centres, particularly across Europe and North America.^73,74^ This may reflect institutional inertia, concerns about technical complexity, or lack of operator familiarity with composite grafting configurations. Moreover, the absence of standardised training pathways for MAG and TAG contributes to variability in practice. The underutilisation of free-IMA grafts represents a missed opportunity to optimise long-term outcomes, especially in younger patients and those with diffuse coronary disease who stand to benefit most from durable arterial conduits.

Importantly, the heterogeneity of study designs and reporting standards in the literature poses challenges for direct comparison and synthesis. Few studies isolate free-IMA outcomes with sufficient granularity, and even fewer report operator-level data or procedural context such as pump status or harvesting technique. This limits the ability to draw definitive conclusions about the superiority of specific configurations. Nonetheless, the consistent signal of safety, patency, and survival benefit across diverse settings supports the continued expansion of free-IMA use, provided that patient selection and technical execution are optimised.

The next phase of research in coronary revascularisation must prioritise prospective, multicentre studies with standardised definitions, procedural transparency, and long-term follow-up. Free-IMA grafting—particularly free-RIMA—has demonstrated promising outcomes, yet its long-term durability beyond 10 years remains insufficiently characterised. High-quality randomised controlled trials are needed to compare free-IMA with RA grafting, evaluate composite vs direct aorto-coronary configurations, and assess sequential vs non-sequential free-IMA strategies. Additionally, the role of free-IMA as a coronary-coronary conduit warrants dedicated investigation, as this technique remains underexplored despite its anatomical and physiological appeal.

These studies should also incorporate surgeon experience, institutional volume, and anatomical complexity into outcome reporting, recognising that technical nuance and operator proficiency significantly influence graft performance. Free-LIMA use, often unplanned and reactive, deserves retrospective and prospective scrutiny to better understand its indications and long-term implications. Establishing the optimal second arterial conduit—if indeed one exists—and defining the most effective arterial configurations for MAG and total arterial grafting TAG will be critical to advancing precision revascularisation.

Ultimately, the knowledge gained from these investigations will inform future guideline development, refine surgical decision-making, and support broader adoption of free-IMA techniques. As the field moves toward tailored, anatomy-driven strategies, the free-IMA graft stands as a versatile and underutilised tool with the potential to reshape long-term outcomes in CABG.

## Conclusion

Maximising arterial grafting remains a vital goal when in-situ LIMA use is unfeasible. Despite robust data supporting multi-arterial and total arterial grafting, these approaches continue to be underutilised in key regions. This gap highlights persistent challenges related to surgical complexity, resource allocation, and training that must be addressed to translate evidence into practice.

Bridging this divide requires a concerted effort to generate compelling, high-quality clinical evidence that confirms the long-term advantages of both in-situ and free arterial grafting techniques, including free-IMA applications. Establishing clear, evidence-based guidelines grounded in such data will empower surgeons to adopt optimal revascularisation strategies with confidence.

Sustained progress in this area is crucial to improving the longevity and effectiveness of coronary bypass surgery, ultimately leading to better patient outcomes and long-term cardiovascular health.
